# Assessing the validity and reliability of self-report data on contraception use in the MObile Technology for Improved Family Planning (MOTIF) randomised controlled trial

**DOI:** 10.1186/s12978-018-0494-7

**Published:** 2018-03-15

**Authors:** Chris Smith, Phil Edwards, Caroline Free

**Affiliations:** 1 Department of Population Health, London School of Hygiene and Tropical Medicine, WC1E7HT, London, UK; 2 Graduate School of Tropical Medicine & Global Health, Nagasaki University, Nagasaki, Japan

**Keywords:** Contraception, Family planning, mHealth, Digital health, Randomised controlled trial

## Abstract

**Background:**

A variety of different approaches to measuring contraceptive use have been used or proposed, either to assess current use or adherence over time, using subjective or objective measures. This paper reports an overview of approaches to measuring adherence to the oral contraceptive, intra-uterine device, sub-dermal implant, and injectable and describes how we assessed contraception use in the MObile Technology for Improved Family Planning (MOTIF) trial in Cambodia.

**Main body:**

We summarise and discuss advantages and disadvantages of different subjective and objective approaches to measuring adherence to the oral contraceptive, intra-uterine device, sub-dermal implant, and injectable such as self-reports, clinic records, electronic monitoring devices, clinical examination and biomarkers.

For the MOTIF trial, we did not consider it feasible to measure objective contraception use as many participants lived a long distance from the clinic and we were concerned whether it was appropriate to ask women to return to clinic for a physical examination simply to verify self-report information already provided.

We aimed to assess the validity of the four-month data with 50 participants, calculating the sensitivity and specificity of self-reported data compared with objective measurement. For the 46 valid measurements obtained, the sensitivity and specificity was 100% for self-reported contraception use compared to objective measurement but this study had some limitations. To assess reliability of self-report data we compared calendar data collected on effective contraception use at months 1–4 post-abortion, collected separately at four and 12 months. Agreement ranged from 80 to 84% with a kappa statistic ranging from 0·59 to 0·67 indicating fair to good agreement.

**Conclusion:**

There is no perfect method of assessing contraception use and researchers designing future studies should give consideration of what to measure, for example current use or detailed patterns of use over time, and remain mindful of what will be feasible and acceptable to the study population. Although self-reported data on contraception use are considered less reliable, and prone to social desirability bias, it is often the standard approach for contraception research and provides data comparable to previous studies.

**Trial registration:**

ClinicalTrials.gov Identifier: NCT01823861. Registered: March 30, 2013.

## Background

Contraception use can be measured for different reasons, for example to collect information on general patterns of contraception use over several years using Demographic and Health Surveys, or to assess the impact of interventions to improve contraception use, often focusing on specific methods, over a shorter time period. A variety of different approaches to measuring contraceptive use have been used or proposed, either to assess current use or adherence over time, using subjective or objective measures. This paper reports an overview of approaches to measuring adherence to the oral contraceptive (OC), intra-uterine device (IUD), sub-dermal implant, and injectable and describes how we assessed contraception use in the MObile Technology for Improved Family Planning (MOTIF) trial in Cambodia [1].

## Main text

### Overview of measuring adherence to common contraceptive methods

The majority of studies assessing contraception use have relied on self-report measures, which are relatively simple and inexpensive to administer. However, concerns about bias associated with self-report measures leading to overestimation of contraceptive use and underestimation of abortion have led to calls for increased rigor in measuring contraception use [2–4].

For fertility surveys, contraceptive calendars, such as those used in Demographic and Health surveys, have been found to generate more complete and accurate data on past self-reported contraceptive use than other questionnaire formats [5–7]. These methods are convenient, inexpensive to administer and the only practical way to obtain information on contraceptive use dating back several years. However, in clinical trials, objective measures are generally considered preferable to subjective measures, as they are less prone to bias [8]. Table 1 summarises approaches to measuring use of different contraceptive methods.

**Table 1:** Summary of approaches to measuring adherence to different contraceptive methods

Contraceptive method	Measurement approach	Advantages	Disadvantages
Oral Contraceptive	Direct observation (clinician observes ingestion of pill)	Accurate and equates to ingestion	Impractical
	Self-reports (self-completed or interview administered questionnaire)	Simple, inexpensive and easy to administer.	Requires training for administrators. Subject to recall and social desirability bias.
	Clinic / pharmacy records	Can help to correct poor recall. Simple, inexpensive and objective. Usually easy to obtain data. Can measure at more than one point in time.	Does not equate to ingestion and requires a closed pharmacy system
	Pill counts (individual pill or pill pack counts)	Objective; quantifiable and easy to perform.	However, easily altered by participant (e.g. pill dumping), cannot assess timing of use
	Electronic Monitoring Devices	Objective, precise, tracks patterns of use over time.	Potentially expensive and may require return visits to download data. Participants may not adhere to using device, intervention might improve use of device rather than pill-taking behaviour.
	Blood hepatic binding globulin levels (Corticosteroid Binding Globulin, Thyroxine Binding Globulin, Lutenizing Hormone and Sex Hormone Binding Globulin)	Objective. Can distinguish between consistent use and non-use (Corticosteroid Binding Globulin and Thyroxine Binding Globulin more discriminating). Inexpensive compared to measuring contraceptive steroid level	Requires specialist laboratory. Can’t distinguish between consistent and inconsistent users
	Blood contraceptive steroid level (e.g. Levonorgestrel or Ethinylestradiol)	Objective, indicates indigestion.	Difficult test, expensive therefore limited potential for replication in other studies, requires a blood test, will not distinguish consistent from inconsistent users
IUD / implant	Self-report	Simple, inexpensive and easy to administer.	Subject to recall bias and social desirability bias
	Clinical examination or ultrasound	Simple, inexpensive and easy to administer.	Intrusive, has to be performed in clinic setting, requires skilled personnel/ equipment
	Clinic or client record	Objective	Requires a closed pharmacy system. Self-held record can get lost
Injection	Self-report	Simple, inexpensive and easy to administer.	Subject to recall bias and social desirability bias.
	Clinic or clinic record	Objective and indicates current use	Requires a closed pharmacy system. Self-held record can get lost

The oral contraceptive is probably the most challenging contraceptive method to measure. The combined oral contraceptive pill (COCP) containing oestrogen and a progestogen is taken for 21 days, with a 7-day break, whereas the progesterone-only pill (POP) containing one hormone is taken continuously. One-year pregnancy rates are estimated to be 0.3% with perfect use, but 7–8% as commonly used [9], thus measures that assess missed pills as well as current use are important.

No gold standard measure of OC use is available. The ideal measure of OC would be one that is objective, can distinguish reliably between non-users, inconsistent, and consistent users, does not rely on a closed pharmacy system (i.e. accounts for people obtaining contraception in different settings); is inexpensive, feasible in different settings, is acceptable to participants, and is applicable to pills containing different hormones. A systematic review of measurement methods for OC use found that the majority (71%) of research studies relied solely on self-report measures (such as interviewer or self-administered questionnaires) rather than objective measures, and the terminology used to describe OC use (such as “continuation”, “compliance” and “adherence”) varied and was rarely described [10].

Objective measures of OC use include biological markers or electronic medication monitors. Biomarkers can either directly measure the hormones in the pill or a proxy measure. Direct measurement of contraceptive steroid levels (for example, Ethinylestradiol or Levonorgestrel) is possible, but only at specialist laboratories, and thus for cost and logistical reasons, might not be feasible in low-income settings. Other researchers have proposed measurement of hepatic binding globulins, which are increased by Ethinylestradiol (EE2). Thyroxine Binding Globulin (TBG) and Corticosteroid Binding Globulin (CBG) were found to distinguish noncompliant users from compliant users [11]. In one study, riboflavin was added to the OC as a urinary marker and assessed by urine florescence [12]. However, the addition of a urine marker to a pill would present significant challenges, and requires a closed pharmacy system. The main advantage of biological measures is that they indicate ingestion of the pill and are good at assessing current use. However, disadvantages are that they are less good at distinguishing inconsistent users from consistent users [10, 13], and blood tests may not be acceptable to study participants. It is theoretically possible to measure EE2 in urine samples by enzyme-linked immunosorbent assay (ELISA), an assay developed for detecting EE2 in animals for the food industry. This might be more acceptable than a blood test, but to our knowledge this test has not been validated for human urine.

Electric monitoring devices (EMDs) can be used to measure adherence to oral medication by recording when a participant opens a pill box or blister pack. Such information can be downloaded periodically or transmitted in real-time. EMDs have been shown to be more accurate than self-report measures, pill counts, and biomarkers for examining antidepressant adherence [14], and used in low income settings, for example to assess anti-retroviral adherence in Kenya [15]. In the field of contraception, two studies reported poorer OC adherence as measured by electronic medication monitoring compared with self-report measures [13, 16]. Thus, advantages of EMDs are that they can provide more detailed information of patterns of use over a period of time compared with self-report. Limitations of EMDs are that it can be difficult to distinguish whether interventions improve pill-taking behaviour or simply improve use of the monitor, that opening the container doesn’t equate to ingestion, and the devices themselves could interfere with the intervention if the participant has to transfer pills into a container. Devices that mimic pill packets would be costly and require a closed pharmacy system.

Measuring use of other contraceptive methods such as implant, IUD and injectable are somewhat easier compared to OC as it is not necessary to assess daily adherence; the women is either protected from pregnancy by the method, or not. These methods can be assessed as follows. First, by self-report (i.e. asking the participant if they are using a method) but this method is subject to biases already mentioned. In particular it might be difficult to recall the date the method was started e.g. date of injection. Second, objectively by reviewing clinic records, but in the case of implant and IUD, this does not indicate continued use if the participant had the device removed in a different clinic. Finally, current use of IUD or implant can be assessed by clinical examination; by palpating the sub-dermal implant or visualising the IUD threads but this entails an intimate examination. IUD or implant use could also be assessed by ultrasound examination.

### Assessing contraception use in the MOTIF trial

For the MOTIF trial, we did not consider it feasible to measure objective contraception use for several reasons. We were concerned that participants wouldn’t be able to return to the clinic for objective follow-up due to lack of time as many lived in rural areas far from the clinic. Furthermore we were concerned about whether it was appropriate, potentially a betrayal of trust, to ask women to return to clinic for internal examination to check IUD threads simply to verify self-report information already provided. We were not able to rely on clinic records of contraception use as the trial was not operating within a closed pharmacy system; oral and injectable contraceptives can be obtained from pharmacists without prescriptions in Cambodia, and long-acting reversible methods (intrauterine device or implant) could be removed in other clinics. Therefore assessment of the primary outcome, use of effective contraception at four and 12 months post-abortion for the MOTIF trial was self-report by phone call. The questionnaire was designed to reduce social desirability bias by first asking participants *“Are you using a contraception method?”* followed by *“Which method are you using?”* if the women answered *“Yes”* (without prompting for specific methods). We felt it was unlikely that participants would over-report using one particular contraception method over another e.g. IUD vs. implant. To assess reliability of self-report data we compared calendar data collected on effective contraception use at months 1–4 post-abortion, collected separately at four and 12 months. Agreement ranged from 80 to 84% with a kappa statistic ranging from 0·59 to 0·67 indicating fair to good agreement.

We aimed to assess the validity of the four-month data with 50 participants, calculating the sensitivity and specificity of self-reported data compared with objective measurement (considered the gold standard) [17]. Consecutive participants recruited from the two peri-urban clinics who had provided self-report follow up were invited to attend for objective assessment of contraceptive use by a research assistant blinded to treatment allocation as follows: assess the position of an implant or IUD (ultrasound or clinical examination according to participant preference); self-held record of injection within the previous three months or permanent method; pill counts defined as > 90% of pills taken since last prescription dispensed. Those attending were given USD$4 for travel expenses. In order to achieve our target of 50 face-to-face objective measurements we attempted to contact 94 participants of contraception use; thus achieving a 53% follow up rate (compared to self-report follow up of 86% at four months). We were unable to contact 18 participants. Twenty participants declined to attend (the most common reason stated was lack of time). Six participants agreed, but then did not attend. We obtained valid measurements in 46 of 50 participants who attended. Three participants did not bring OC with them and one was ineligible as she had not provided self-reported contraception data. Amongst these 46 participants, the sensitivity and specificity was 100% for self-reported IUD, implant, injectable and OC use compared to objective measurement (Table 2).

**Table 2:** Objective vs. self-report follow up at four months

	Self-report (number)	Objective measurement (number)
Current contraception use		
Intra-uterine device	11	11
Implant	4	4
Injectable	3	3
Oral contraceptive	9	9
No method/non-effective method	19	19
Total effective	27	27
Total no/non-effective method	19	19
Total measurements included in analysis	46	46

Amongst the 46 participants the sensitivity of self-report data compared to objective measurement (gold standard) was 100% (27/27*100) and the specificity was 100% (19/19*100)

It is uncertain if this validity study was helpful. Whilst the sensitivity and specificity of objectively measured contraception was 100% in those attending follow-up, the response rate was low and it is not clear that those who attended were representative of the wider study population. For example, if a participant provided inaccurate self-report data, they might be less likely to attend for objective assessment.

## Conclusion

In the MOTIF trial, use of self-report measures resulted in higher rates of follow up compared to attempts at objective measures. Self-report measures showed fair/good reliability and the validity study did not identify any cases of misclassification. However, the method for verifying self-reported OC use by pill counts was also prone to detection bias.

There is no perfect method of assessing contraception use and researchers designing future studies should give consideration of what to measure, for example current use or detailed patterns of use over time, and remain mindful of what will be feasible and acceptable to the study population. For OC use, researchers should consider using the definitions of ‘continuation’, ‘discontinuation’, ‘interrupted use’ and ‘missed pills’ as recommended by Hall et al. [10].

Objective measures using clinic records or electronic medication monitors might be possible if there is a closed pharmacy system. EMDs linked to mobile devices providing real-time data on pill taking will provide the most detailed information on OC use. Biological measures or clinical examination might be feasible if participants are willing and able to return to the clinic, and there is laboratory capacity. Self-report measures can be optimised by careful consideration of questions to avoid response-style bias. Although self-reported data on contraception use are considered less reliable, and prone to social desirability bias, it is often the standard approach for contraception research and provides data comparable to previous studies [2, 10]. A validity study could be considered to verify self-report measures. Future research could explore the possibility of a urine EE2 assay as an alternative to a blood test to distinguish between users and non-users of OC.

## Abbreviations

CBG: Corticosteroid Binding Globulin
COCP: Combined oral contraceptive pill
EE2: Ethinylestradiol
ELISA: Enzyme-linked immunosorbent assay
EMD: Electric monitoring devices
IUD: Intra-uterine device
MOTIF: MObile Technology for Improved Family Planning
OC: Oral contraceptive
POP: Progesterone-only pill
TBG: Thyroxine Binding Globulin
USD: United States Dollar
